# Supplementary material for: Study of Membrane-Immobilized Oxidoreductases in Wastewater Treatment for Micropollutants Removal
Source: Int J Mol Sci. 2022 Nov 15;23(22):14086. doi: 10.3390/ijms232214086 (PMC9699638; doi:10.3390/ijms232214086)
Supplement: Supplementary file 1 [file ijms-23-14086-s001.zip › ijms-1942594-SI.pdf]

International Journal of *Molecular Sciences*

MDPI

Supplementary materials

S1. Composition of wastewater "before"

Table S1. Various substances detected in wastewater "before" obtained from local wastewater treatment plant using GC-MS analysis.

Detected compound
(Z)-4-Methyl-2-hexene
Azelaic acid
Butane, 2,2,3,3-tetramethyl-
1-Pentanol, 2-ethyl-4-methyl-
Hexane, 2,5-dimethyl-
Cyclopentane, 1,3-dimethyl-, cis-
Hexane, 2,4-dimethyl-
Cyclohexane, methyl-
Palladium(0), bis(ü-2-butadiene) 1,1,4,5,8,8-hexa(tert.-butyl)-1,4,5,8-tetraphosphaoctane
Cyclopentane, butyl-
Hexane, 3,3-dimethyl-
Isopropylcyclobutane
Pentane, 2,3,4-trimethyl-
Valeric anhydride
Hexane, 2,3-dimethyl-
Pentane, 3-ethyl-2-methyl-
Heptane, 2-methyl-
Hexane, 3,4-dimethyl-
Heptane, 3-methyl-
Hexane, 3-ethyl-
1,3,5-Cycloheptatriene
Pentane, 2,2,4-trimethyl-
1,1':3',1''-Terphenyl, 3,3'',5,5''-tetrabromo-5-(3,5-dibromophenyl)-

Cyclopentane, 1-ethyl-3-methyl-, trans-
Hexane, 2,3,4-trimethyl-
Hexyl alcohol
Hexane, 3-ethyl-
Octane, 3,4-dimethyl-
Antazoline
p-Xylene
Nonane
Ethylbenzene
Octane, 2,7-dimethyl-
Propane, 2-methyl-1-nitro-
Octane, 3,4-dimethyl-
Molybdenum, bis[(1,2,3,4,5-ü)-1,3-bis(1,1-dimethylethyl)-2,4-cyclopentadien-1-yl]di-æ-carbonyldicarbonyldi-
Nonane, 2-methyl-
2,4,4-Trimethyl-1-pentanol
Hexane, 3-ethyl-2,5-dimethyl-
Hematoporphyrin
1,3-Propanediol, tert-butyldimethylsilyl ether
2-Butene-1,4-diol
Decane
Heptane, 3-ethyl-5-methyl-
Hexane, 4-ethyl-2-methyl-
Heptane, 3-ethyl-5-methyl-
Octane, 2,3,6,7-tetramethyl-
Decane, 3,7-dimethyl-
Heptane, 4-ethyl-
Decane, 2,4-dimethyl-
Octane, 3,5-dimethyl-
6-Methyl-2-heptanol
Octane, 2-methyl-
Undecane, 2-methyl-
Octane
Octane, 2,4,6-trimethyl-
Tridecane
Undecane, 2,4-dimethyl-
Dodecane, 6-methyl-
Heptane, 3,3-dimethyl-
Octane, 2,4,6-trimethyl-
2-Propyl-1-pentanol
Dodecane, 4,6-dimethyl-
Benzaldehyde, 2,5-dimethyl-
Ether, hexyl pentyl
Benzene, 1,3-bis(1,1-dimethylethyl)-
Sulfurous acid, 2-ethylhexyl hexyl ester
1-Octanol
Hydroxylamine, O-decyl-
Heptane, 3-ethyl-5-methyl-
Hexane, 3,3-dimethyl-
Decane, 6-ethyl-2-methyl-
Sulfurous acid, 2-propyl undecyl ester
Decane, 6-ethyl-2-methyl-

Valproic Acid
2-Propyl-1-pentanol
Tetradecane
Oxalic acid, allyl tridecyl ester
Heptane, 2,2,3,3,5,6,6-heptamethyl-
Octane, 3,3-dimethyl-
3-Hexanone, 2,4-dimethyl-
Undecane, 4,7-dimethyl-
Undecane, 2,4-dimethyl-
Pentane, 1-butoxy-
Tetratetracontane
Tetracycline
Hexadecane
Decane, 6-ethyl-2-methyl-
Phenol, 3,5-bis(1,1-dimethylethyl)-
3-Ethyl-3-methylheptane
17α-Ethynylestradiol
2-Decanol
Octane, 2,4,6-trimethyl-
2-Ethyl-1-hexanol
tert-Amyl alcohol
Sulfurous acid, 2-ethylhexyl hexyl ester
Dodecane, 2,6,11-trimethyl-
Hexane, 3,3-dimethyl-
Octane, 2,4,6-trimethyl-
Sulfurous acid, 2-ethylhexyl hexyl ester
Heptadecane
3-Ethyl-3-methylheptane
Monopalmitin
Propanoic acid, 2-methyl-, 2-ethyl-1-propyl-1,3-propanediyl ester
Pentadecanal-
2-Decanol
1,1':3',1''-Terphenyl, 3,3'',5,5''-tetrabromo-5-(3,5-dibromophenyl)-
Decane, 2,4,6-trimethyl-
Dodecane, 2,7,10-trimethyl-
Pentacontanoic acid, ethyl ester
Decane, 2,9-dimethyl-
Decane, 6-ethyl-2-methyl-
1-(2-Methoxyethoxy)-2-methyl-2-propanol
Ketoprofen methyl ester
Pentadecanal-
Tridecane, 1-iodo-
Hexadecane
1-Decanol, 2-hexyl-
Acetic acid, hydrazide
Phytol
Sulfurous acid, 2-ethylhexyl hexyl ester
2-Hydroxy-2,4-dimethyl-3-pentanone
(2,4-Dichlorophenoxy)acetic acid
Tetratetracontane

S2. Composition of wastewater "after"

Table S2. Various substances detected in wastewater "before" obtained from local wastewater treatment plant using GC-MS analysis.

Detected compound
Butane, 2,2,3,3-tetramethyl-
Hexane, 2,5-dimethyl-
Cyclopentane, 1,2-dimethyl-, cis-
Hexane, 2,4-dimethyl-
4-Hexen-1-ol
Cyclohexane, methyl-
Propane, 2-methyl-1-nitro-
Cyclopentane, ethyl-
Hexane, 3,3-dimethyl-
Cyclopropane, 1,2-dimethyl-, trans-
Pentane, 2,3,4-trimethyl-
1-Hexene, 4-methyl-
Hexane, 2,3-dimethyl-
Heptane, 2-methyl-
Pentane, 3-ethyl-
Hexane, 3,4-dimethyl-
Heptane, 3-methyl-
Toluene
Pentane, 2,2,4-trimethyl-
Cyclopentane, 1-ethyl-3-methyl-, trans-
Octane
Hexane, 2,3,5-trimethyl-
Heptane, 2,4-dimethyl-
Valproic Acid
Octane, 4-methyl-
Ethylbenzene
p-Xylene
Nonane
Ethylbenzene
Octane, 2,7-dimethyl-
Hematoporphyrin
Pentane, 3-ethyl-2,4-dimethyl-
Heptane, 3-ethyl-
Hexane, 3-ethyl-2,5-dimethyl-
2-Propyl-1-pentanol
Decane
1,3-Propanediol, tert-butyldimethylsilyl ether
Heptane, 3,3,4-trimethyl-
Decane, 2,4-dimethyl-
Hexane, 4-ethyl-2-methyl-
Nonane, 2,6-dimethyl-
Decane, 3,7-dimethyl-
Heptane, 4-ethyl-
Octane, 2-methyl-
Octane, 2,7-dimethyl-
Citraconic acid

N-Isovaleroylglycine
1,1':3',1''-Terphenyl, 3,3'',5,5''-tetrabromo-5-(3,5-dibromophenyl)-
Dodecane
Undecane, 2,4-dimethyl-
Heptane, 4-propyl-
Undecane, 2,8-dimethyl-
Octane, 2,4,6-trimethyl-
1,1':3',1''-Terphenyl, 3,3'',5,5''-tetrabromo-5-(3,5-dibromophenyl)-
Decane, 2,5,9-trimethyl-
Benzaldehyde, 2,5-dimethyl-
Ether, hexyl pentyl
Benzene, 1,3-bis(1,1-dimethylethyl)-
10-Methylnonadecane
Hexadecane
Isobutyl 3-methylbutyl disulfide
1,3-Hexanediol, 2-ethyl-
(2,4-Dichlorophenoxy)acetic acid
Oxalic acid, isohexyl neopentyl ester
Octane
2,6-Dimethyldecane
Pyridine, 3-(1-methyl-2-pyrrolidinyl)-, (S)-
Tetradecane
Oxalic acid, allyl decyl ester
Oxalic acid, allyl nonyl ester
Heptane, 3,3-dimethyl-
Octane, 2,4,6-trimethyl-
Decane, 6-ethyl-2-methyl-
Sulfurous acid, decyl 2-propyl ester
Decane, 2,4-dimethyl-
Hexadecane
Octane, 2,4,6-trimethyl-
tert-Amyl alcohol
2,2,4-Trimethyl-3-pentanone
Phenol, 2,4-bis(1,1-dimethylethyl)-
Hexane, 3,3-dimethyl-
9-methylheptadecane
Sulfurous acid, 2-ethylhexyl hexyl ester
Sulfurous acid, butyl isohexyl ester
Undecane, 2-methyl-
1,1':3',1''-Terphenyl, 3,3'',5,5''-tetrabromo-5-(3,5-dibromophenyl)-
2-Heptadecenal
17α-Ethynylestradiol
Decane, 6-ethyl-2-methyl-
Sulfurous acid, 2-ethylhexyl hexyl ester
1,1':3',1''-Terphenyl, 3,3'',5,5''-tetrabromo-5-(3,5-dibromophenyl)-
3-Hepten-1-ol
Sulfurous acid, 2-ethylhexyl hexyl ester
2-Propyl-1-pentanol
Decane, 3-ethyl-3-methyl-
Octadecane, 2-methyl-
Tetracycline

Oxalic acid, isohexyl pentyl ester
Molybdenum, bis[(1,2,3,4,5-ü)-1,3-bis(1,1-dimethylethyl)-2,4-cyclopentadien-1-yl]di-æ-carbonyldicarbonyldi-
Pentadecanal-
Undecane, 2,6-dimethyl-
Sulfurous acid, 2-ethylhexyl hexyl ester
Ketoprofen methyl ester
Decane, 3-ethyl-3-methyl-
1,1':3',1''-Terphenyl, 3,3'',5,5''-tetrabromo-5-(3,5-dibromophenyl)-
á-Hydroxyisovaleric acid
Propanoic acid, 2-oxo-, trimethylsilyl ester
3-Hexanone
Heptacosane
Malonamic acid
Oxalic acid, allyl nonyl ester
Sulfurous acid, 2-ethylhexyl isohexyl ester

Table S3. Removal efficiency of hematoporphyrin, (2,4-dichlorophenoxy)acetic acid (2,4-D), 17α-ethynylestradiol (EE2), tetracycline, tert-amyl alcohol and ketoprofen methyl ester after treatment of wastewater "before" by various biocatalytic membranes. **1** denotes rejection by pristine membrane, **2** denotes removal rate by membrane with inactivated enzyme, **3** denotes removal rate by catalytic conversion, **4** denotes total removal rate of micropollutant.

**Analyzed compound**	**Membrane-laccase**				**Membrane-tyrosinase**				**Membrane-HRP**			
	**1**	**2**	**3**	**4**	**1**	**2**	**3**	**4**	**1**	**2**	**3**	**4**
Hematoporphyrin	3%	5%	13%	**21%**	3%	4%	3%	**10%**	3%	3%	5%	**11%**
(2,4-Dichlorophenoxy)acetic acid	10%	11%	30%	**51%**	10%	12%	23%	**45%**	10%	8%	2%	**20%**
17α-Ethynylestradiol	7%	5%	30%	**42%**	7%	3%	30%	**40%**	7%	4%	4%	**15%**
Tetracycline	6%	14%	28%	**48%**	6%	9%	21%	**36%**	6%	7%	8%	**21%**
tert-Amyl alcohol	4%	2%	23%	**29%**	4%	3%	14%	**21%**	4%	2%	3%	**9%**
Ketoprofen methyl ester	8%	9%	20%	**37%**	8%	8%	11%	**27%**	8%	5%	5%	**18%**

Table S4. Removal efficiency of hematoporphyrin, (2,4-dichlorophenoxy)acetic acid (2,4-D), 17α-ethynylestradiol (EE2), tetracycline, tert-amyl alcohol and ketoprofen methyl ester after treatment of wastewater "after" by various biocatalytic membranes. **1** denotes rejection by pristine membrane, **2** denotes removal rate by membrane with inactivated enzyme, **3** denotes removal rate by catalytic conversion, **4** denotes total removal rate of micropollutant.

**Analyzed compound**	**Membrane-laccase**				**Membrane-tyrosinase**				**Membrane-HRP**			
	**1**	**2**	**3**	**4**	**1**	**2**	**3**	**4**	**1**	**2**	**3**	**4**
Hematoporphyrin	3%	8%	15%	**26%**	3%	6%	4%	**13%**	3%	3%	5%	**11%**
(2,4-Dichlorophenoxy)acetic acid	10%	13%	38%	**61%**	10%	17%	22%	**49%**	10%	10%	4%	**24%**
17α-Ethynylestradiol	7%	6%	36%	**49%**	7%	6%	33%	**46%**	7%	6%	4%	**17%**
Tetracycline	6%	18%	32%	**56%**	6%	12%	20%	**38%**	6%	8%	12%	**26%**
tert-Amyl alcohol	4%	4%	22%	**30%**	4%	5%	18%	**27%**	4%	4%	5%	**13%**
Ketoprofen methyl ester	8%	13%	19%	**40%**	8%	11%	13%	**32%**	8%	8%	3%	**19%**
